# The Molecular Targets and Anti-Invasive Effects of 2,6-bis-(4-hydroxyl-3methoxybenzylidine) cyclohexanone or BHMC in MDA-MB-231 Human Breast Cancer Cells

**DOI:** 10.3390/molecules23040865

**Published:** 2018-04-10

**Authors:** Siti Nor Aini Harun, Daud Ahmad Israf, Chau Ling Tham, Kok Wai Lam, Manraj Singh Cheema, Nur Fariesha Md Hashim

**Affiliations:** 1 Department of Biomedical Sciences, Faculty of Medicine and Health Sciences, Universiti Putra Malaysia, Serdang 43400, Selangor, Malaysia; sitinorainiharun@gmail.com (S.N.A.H.); daudaia@upm.edu.my (D.A.I.); chauling@upm.edu.my (C.L.T.); manraj@upm.edu.my (M.S.C.); 2 Faculty of Pharmacy, Universiti Kebangsaan Malaysia, Jalan Raja Muda Abdul Aziz, Kuala Lumpur 50300, Malaysia; david_lam_98@yahoo.com

**Keywords:** BHMC, breast cancer invasion, invadopodia, β-PIX, MMP-9, MT1-MMP

## Abstract

In order to metastasize, tumor cells need to migrate and invade the surrounding tissues. It is important to identify compound(s) capable of disrupting the metastasis of invasive cancer cells, especially for hindering invadopodia formation, so as to provide anti-metastasis targeted therapy. Invadopodia are thought to be specialized actin-rich protrusions formed by highly invasive cancer cells to degrade the extracellular matrix (ECM). A curcuminoid analogue known as 2,6-bis-(4-hydroxy-3-methoxybenzylidine)cyclohexanone or BHMC has shown good potential in inhibiting inflammation and hyperalgesia. It also possesses an anti-tumor effects on 4T1 murine breast cancer cells in vivo. However, there is still a lack of empirical evidence on how BHMC works in preventing human breast cancer invasion. In this study, we investigated the effect of BHMC on MDA-MB-231 breast cancer cells and its underlying mechanism of action to prevent breast cancer invasion, especially during the formation of invadopodia. All MDA-MB-231 cells, which were exposed to the non-cytotoxic concentrations of BHMC, expressed the proliferating cell nuclear antigen (PCNA), which indicate that the anti-proliferative effects of BHMC did not interfere in the subsequent experiments. By using a scratch migration assay, transwell migration and invasion assays, we determined that BHMC reduces the percentage of migration and invasion of MDA-MB-231 cells. The gelatin degradation assay showed that BHMC reduced the number of cells with invadopodia. Analysis of the proteins involved in the invasion showed that there is a significant reduction in the expressions of Rho guanine nucleotide exchange factor 7 (β-PIX), matrix metalloproteinase-9 (MMP-9), and membrane type 1 matrix metalloproteinase (MT1-MMP) in the presence of BHMC treatment at 12.5 µM. Therefore, it can be postulated that BHMC at 12.5 µM is the optimal concentration for preventing breast cancer invasion.

## 1. Introduction

Breast cancer is one of the most common cancers affecting women worldwide [1]. Based on records from the National Cancer Institute of Malaysia, breast cancer leads the overall cancer cases by 31.1% from 2007–2011 [2]. The huge percentage of cases makes it the leading factor that contributes to cancer mortality in Malaysia. Breast cancer is well-known for its ability to spread to other parts of the body. This is known as cancer metastasis [3]. Common parts of the body where breast cancer can spread are liver, lungs, the brain, and bones [3]. Among all the breast cancer cases, triple negative breast cancer (TNBC) is the highest grade found in solid breast tumors. TNBC is the most difficult case to treat since the cancer cells lack receptors related to the progression of breast cancer such as estrogen, progesterone, and HER2 expression [4].

Metastatic breast cancer is a multistep process that starts with the escape of tumor cells from the primary tumor, degradation and invasion through the extracellular matrix (ECM), entry into the vasculature and dissemination to distant sites [5]. In order to degrade and invade the ECM, highly invasive cancer cells are thought to form specialized membrane protrusions termed invadopodia [6]. Invadopodia are formed as finger-like projections possesed by cancer cells with extensive remodeling of the actin cytoskeleton that appear in the direction of cell movement [7]. These protrusions possess degrading activities, which is one of the prerequisites of the first step of metastatic cascade [8]. In recent years, the formation of invadopodia has extensively been explored to discover the proteins involved [9]. Studies have found that invadopodia are the main control in organizing the steps in metastasis comprising many proteins such as cortactin, Actin-related protein 2/3 (Arp 2/3), Neural Wiskott-Aldrich syndrome protein (N-WASP), MMP-9, MT1-MMP, adapter protein Tks4, adapter protein Tks5, Ezrin, profilin, β-PIX, and many more [10,11,12]. Each of these proteins play a different role but some are related to each other, which is either to initiate the actin reorganization or to degrade the ECM [11,13,14]. Recent reports have suggested the possible pathways connecting these proteins in order to comprehensively translate the investigation into useful drug production studies [13,15]. Studies also use computer simulations to observe the synergistic effect on blocking cancer invasion by blocking MT1-MMPs alone and a combination of blocking several components of MMPs, in which the effect is more on hindering invasion [16].

Metastatic breast cancer is a major problem, as it can affect the survival of cancer patients [17]. Recently, the treatment itself has introduced the recurrence of cancer besides producing side effects and promoting invasion and metastasis [18,19]. Therefore, new exploration into the compounds or drugs to serve as chemoprevention should be vigorously done [17].

Curcumin, a diarylheptanoid compound found in turmeric, has been studied for many years and has been proven to show positive outcomes as an anti-bacterial [20], anti-malarial [21], anti-oxidant [22], anti-inflammation [23] and anti-cancer compound [24,25,26]. Nevertheless, due to its poor bioavailability, researchers have adopted many approaches to overcome this issue, including encapsulating the curcumin as a nanoparticle [20] and synthesizing analogues of curcumin [27]. Our group synthesized an analogue of curcumin known as 2,6-bis-(4-hydroxy-3-methoxybenzylidine) cyclohexanone or BHMC. The synthesis of BHMC was devised by referring to the structure of curcumin. The β-diketone moiety of the curcumin was eliminated and modified into a conjugated double bond while maintaining the phenolic OH group for its anti-oxidant properties [27]. This curcuminoid analogue was studied in inflammation [27], sepsis [28], hyperalgesia [29] and murine breast cancer [30]. BHMC has been shown to inhibit the pro-inflammatory cytokines, which are the monocyte chemotactic protein (MCP)-1, interleukin (IL)-10, and tumor necrosis factor (TNF)-α [27]. BHMC also inhibits the signaling pathway of inflammation, which consists of a nuclear factor (NF-κB), activator protein (AP)-1 and p-38 and mitogen-activated protein kinase (MAPK). It also protects from lethal sepsis in the caecal-ligation puncture (CLP) model of severe sepsis [28]. Besides that, BHMC was also proven to elicit an anti-hyperalgesic effect in the neuropathic pain model in mice [29]. Recently, BHMC has been studied on murine 4T1 breast cancer cells [30]. That particular study showed that BHMC reduced the number of mitotic cells in 4T1-challenged mice, which is related to some anti-proliferative effect possessed by BHMC. Besides that, the metastasis of 4T1 cells to the lung was also reduced, suggesting the anti-metastatic effect possessed by BHMC [30]. Thus it is interesting to observe the effect of BHMC on MDA-MB-231 human breast cancer cells.

In the present study, we showed that BHMC significantly prevented cancer invasion, which is one of the key steps of the metastasis of breast cancer. This action appears to be due to the inhibition of invadopodia formation and the inhibition of invadopodia-related proteins, which are β-PIX, MMP-9, and MT1-MMP. Our results provide new direction for BHMC treatment in that it may interrupt the progression of breast cancer.

## 2. Results

### 2.1. Cytotoxicity and Effects of BHMC on the Proliferation of MDA-MB-231 Cells

Determination of the concentration of BHMC is important to check the cytotoxicity of BHMC on MDA-MB-231 cells. A MTT assay was used to determine the effect of BHMC on MDA-MB-231 cell metabolic activity. The concentrations of BHMC used in the MTT assay were in the range of 1.6 to 100 µM. 

**Figure 1:**
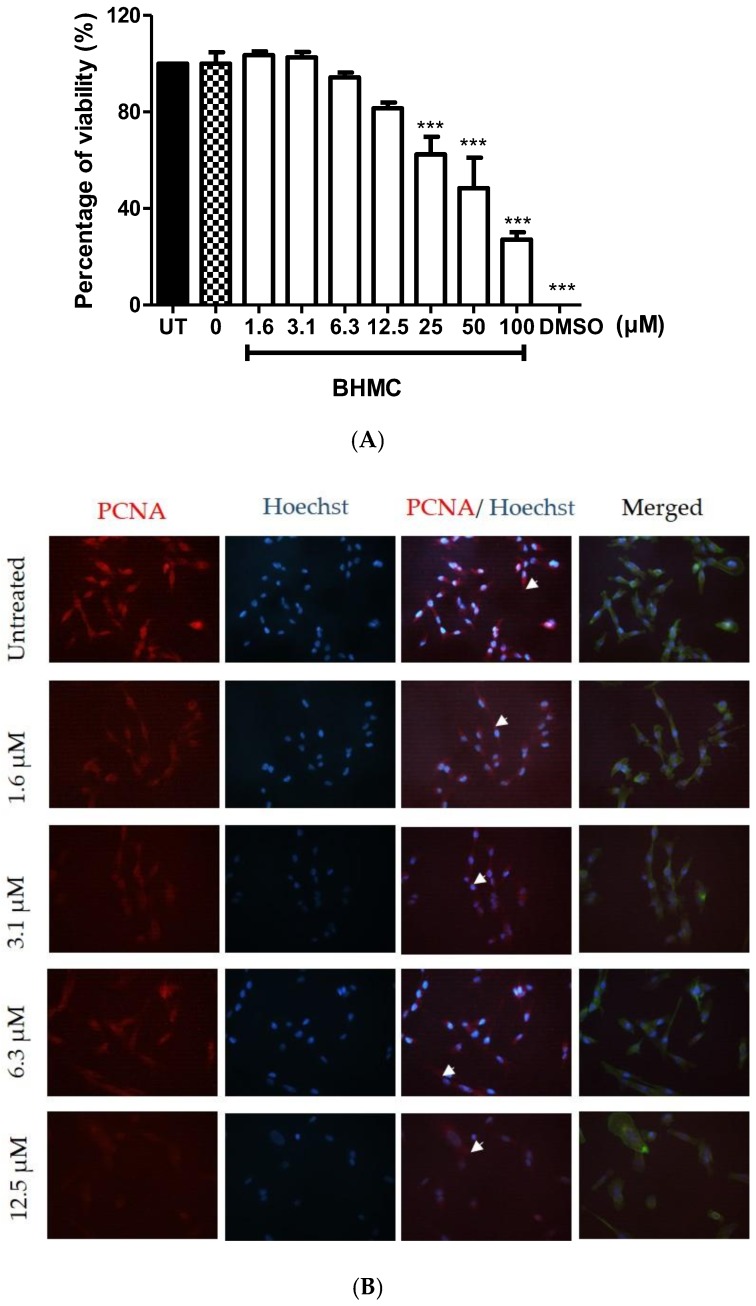

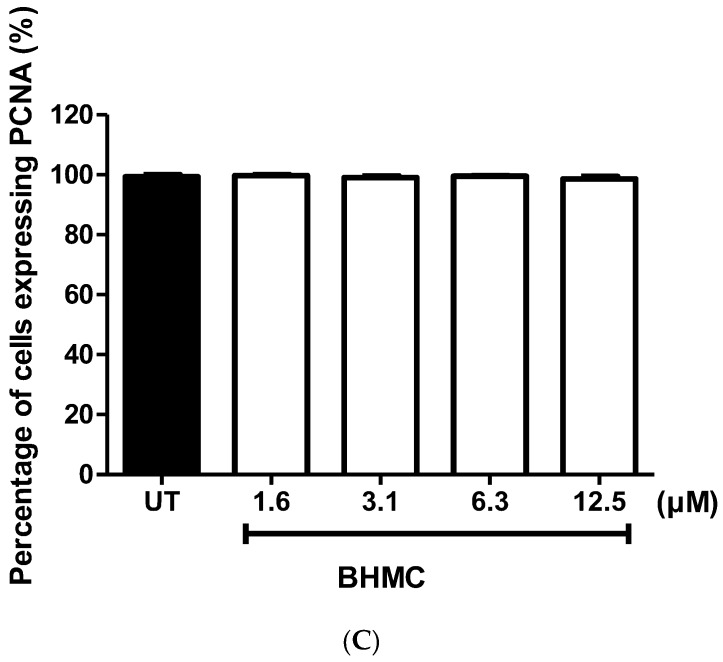
Cell viability and cell proliferation assays. (**A**) MDA-MB-231 cells (1 × 10^5^ cells/mL) were treated with different concentrations of BHMC for 24 h and detected for cell viability using MTT assay. (**B**) MDA-MB-231 cells (2 × 10^4^ cells/mL) were fixed and stained with anti-PCNA, Alexa Fluor 568 goat anti-mouse secondary antibody (red), Hoechst (blue), and Alexa Fluor 488 phalloidin (green) after treating with BHMC for 24 h using indicated concentrations (mag. 400×). The arrows indicate the presence of PCNA in the cytoplasm of the cells. (**C**) The graph is plotted to represent the presence of PCNA in 150 MDA-MB-231 cells for each condition. All values are the mean ± S.E.M of three independent experiments. *** *p* < 0.001, which is significantly different from the untreated group.

Figure 1A shows the effect of BHMC on MDA-MB-231 cell metabolic activity or cell viability in this study. Treatment with BHMC reduced the cell viability at 25 µM and above on MDA-MB-231 cells. Thus, concentrations of BHMC at 12.5 µM and below were used for subsequent assays to avoid interference resulting from the anti-proliferative effect of BHMC. Treatment of BHMC at 12.5 µM and below was further checked for cell proliferation with the presence of proliferating cell nuclear antigen (PCNA) antibody. All cells counted in all conditions of BHMC ranging from 1.6 to 12.5 µM expressed PCNA. These are then stained with an antibody for visualization purposes (Figure 1B,C) including the control group.

### 2.2. Inhibition of BHMC on the Migration and Invasion of MDA-MB-231 Cells

**Figure 2:**
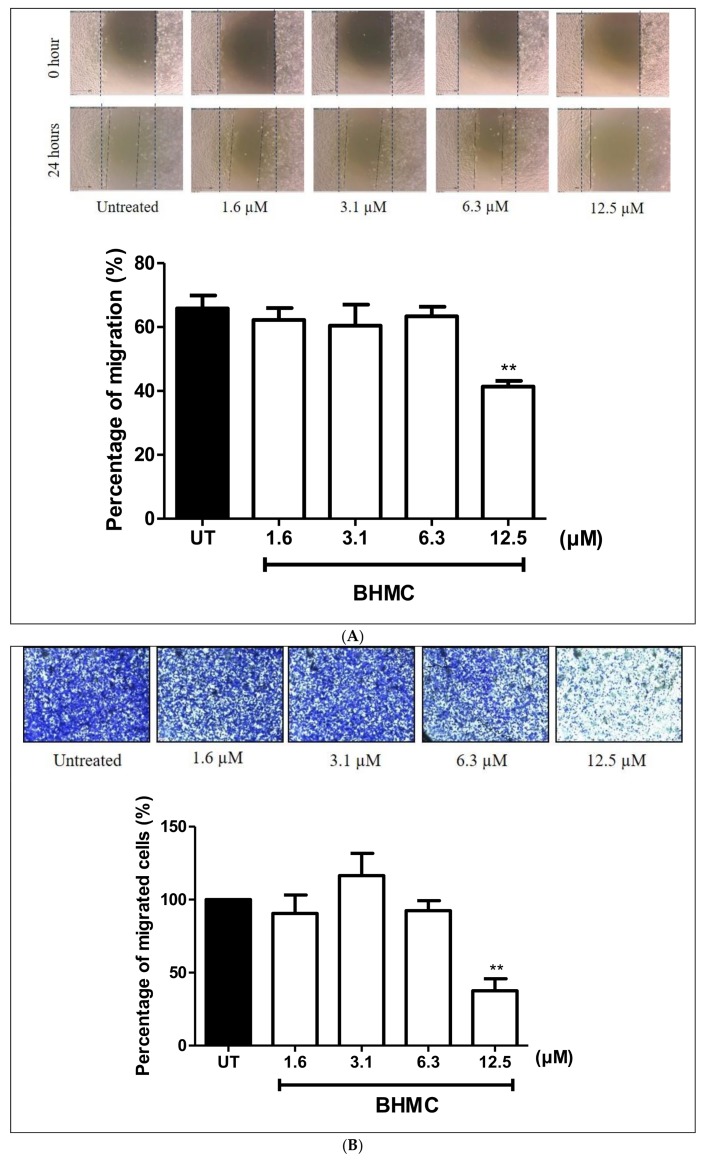

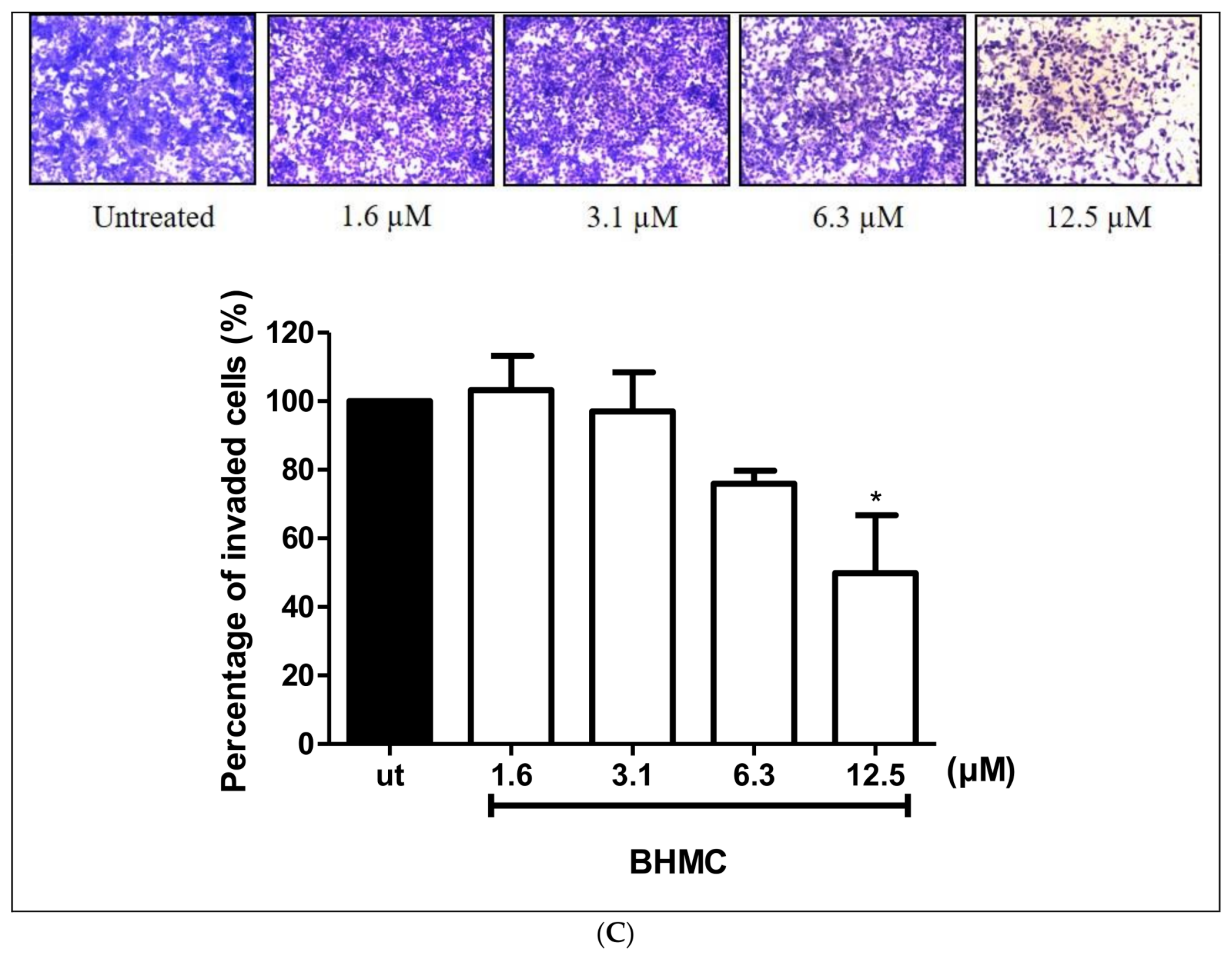
Effects of BHMC on the migration and invasion of MDA-MB-231 cells using scratch migration assay, transwell migration, and transwell invasion assays. (**A**) Confluent MDA-MB-231 cells were wounded with a vertical pipette tip and treatment of BHMC of indicated concentrations were added for 24 h. The cells were photographed under inverted microscopy at 0 h and at 24 h. The distance the cells migrated were calculated and converted into a percentage. The outer dotted line is the mark of the distance at 0 h while the black line is the mark of distance at 24 h. (**B**) MDA-MB-231 cells were seeded into 8 µm transwell inserts and treated with indicated concentrations of BHMC for 24 h. The cells were stained with 0.2% crystal violet. The images were captured at five different fields using a magnification of 100X. The stained cells were lysed with 100% acetic acid and absorbance was measured at 570 nm. (**C**) For transwell invasion, the MDA-MB-231 cells seeded on rat-tail collagen type I in 8 µm inserts were treated with the indicated concentrations of BHMC for 24 h. The cells were then stained with 0.2% crystal violet. The images were captured at five different fields using a magnification of 100X. Then, the dye was lysed with 100% acetic acid and the absorbance was measured at 570 nm. The data represents the mean ± S.E.M of three independent experiments. * *p* < 0.05 and ** *p* < 0.01, which is significantly different from the untreated group.

Migration and invasion are important steps in cancer metastasis [31]. Scratch migration assay, transwell migration, and transwell invasion assays were used to investigate the effect of BHMC on the migration and invasion of MDA-MB-231 cells. Treating the cells with BHMC at 12.5 µM (*p* < 0.01) significantly decreased the migration of MDA-MB-231 cells (Figure 2A). This was confirmed with the results of the transwell migration assay (Figure 2B) in comparison to the untreated group. The transwell migration assay (Figure 2B) shows that BHMC reduced the cell numbers that migrated through the inserts. We also tested the ability of MDA-MB-231 cells to invade the matrix using the transwell invasion assay upon treatment with BHMC. Treatment of BHMC significantly reduced (*p* < 0.05) the number of invaded cells at 12.5 µM; this is consistent with previous assays (Figure 2C). These findings prove that BHMC prevents the migration and invasion of MDA-MB-231 cells.

### 2.3. BHMC Effects on the Number of Cells Forming Invadopodia

**Figure 3:**
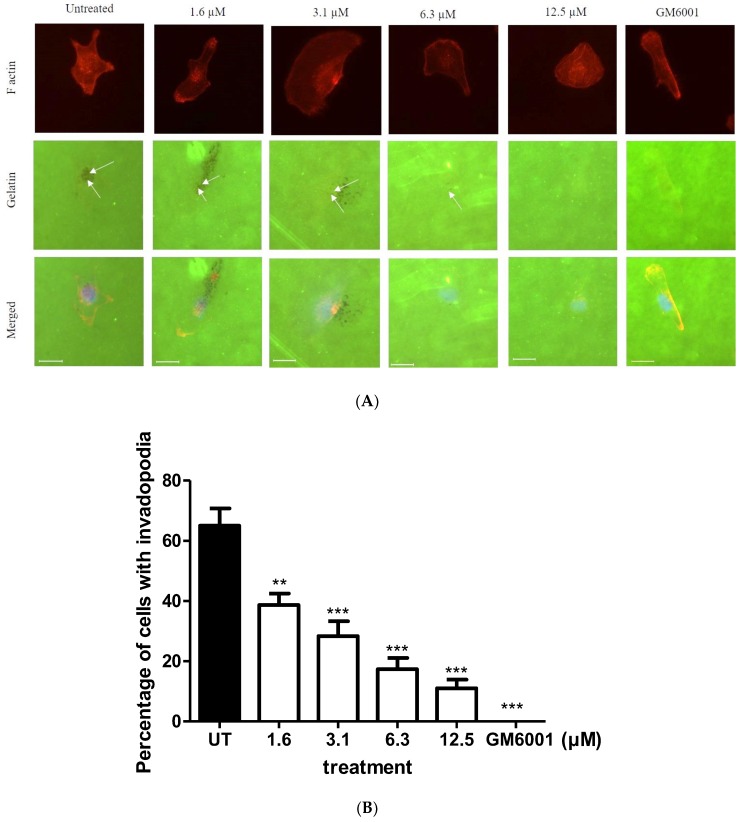
Effects of BHMC on gelatin degradation assay. (**A**) Cells seeded in a 6-well plate (5 × 10^4^ cells/mL) were treated with indicated concentrations of BHMC for 24 h and then re-seeded (2 × 10^4^ cells/mL) on Oregon Green 488 gelatin-coated coverslips for 3 h, fixed and stained for F-actin. Images were captured at a magnification of 400X. Arrows indicate the degradation on OG 488 gelatin. (**B**) Cells were scored for the presence of actin spots with underlying gelatin degradation for 100 cells for each condition. In all cases, the results are the mean ± S.E.M of three independent experiments. ** *p* < 0.01 and *** *p* < 0.001, which is significantly different from the untreated group. Scale bar = 20 µm.

MDA-MB-231 cells have been extensively studied for their potential to successfully form invadopodia when they are placed on a matrix [14,32]. Invadopodia have a dot-like appearance with an actin-rich core in a 2D matrix degradation assay [14]. These dots are the accumulation of many proteins, assembled together to perform their own functions and producing small punctate finger-like projections near the cell nucleus that extend proteolytically into the matrix [14]. We tested the ability of MDA-MB-231 to form invadopodia on Oregon Green 488 gelatin-coated coverslips upon BHMC treatment and found that BHMC reduces the number of cells that form invadopodia in a concentration-dependent manner (Figure 3A,B). GM6001, an MMP-inhibitor, was used to synchronize the formation of invadopodia appearing in MDA-MB-231 cells. There is an absence of invadopodia in all cells upon treatment with GM6001 at 10 µM.

### 2.4. Inhibition of β-PIX, MMP-9, and MT1-MMP Protein Expression on MDA-MB-231 Cells upon BHMC Treatment

**Figure 4:**
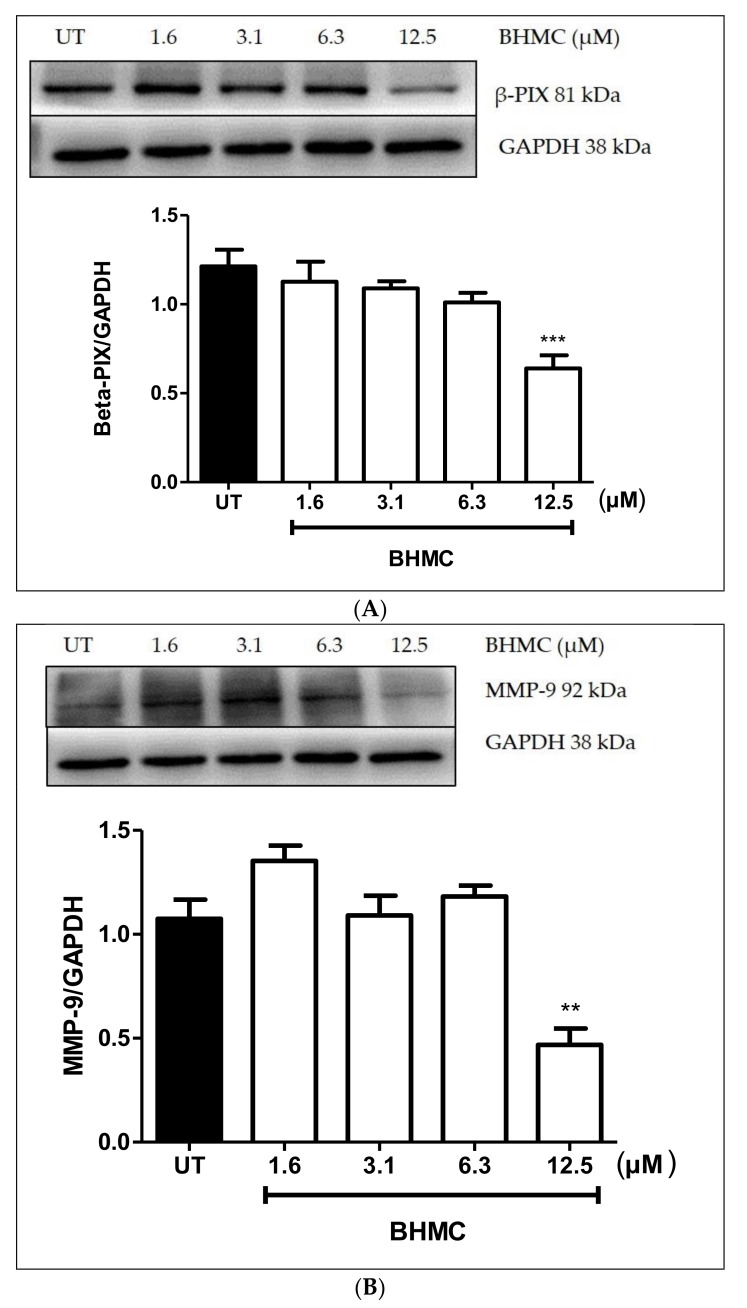

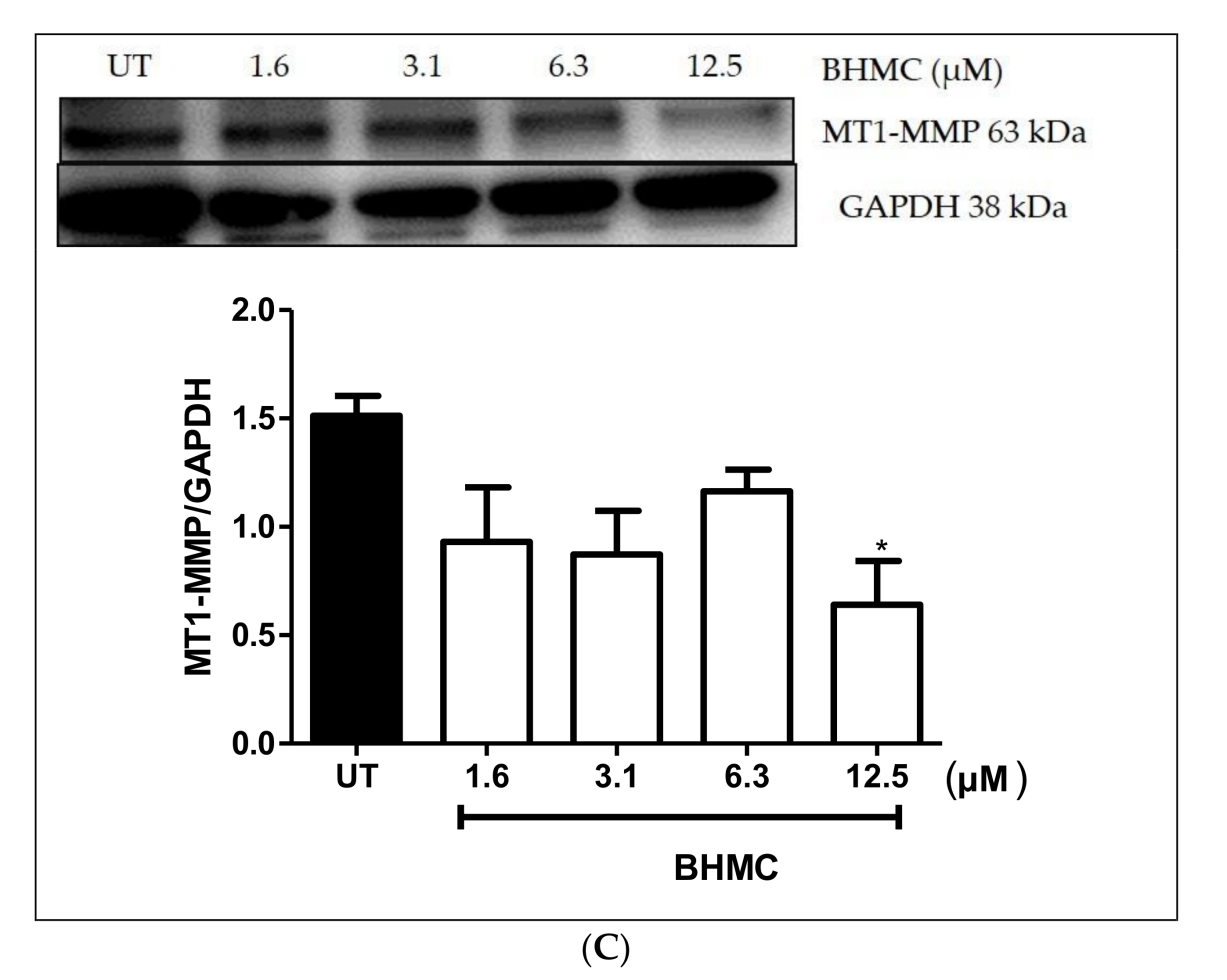
Effects of BHMC on β-PIX, MMP-9, MT1-MMP, protein expression. MDA-MB-231 cells were treated with indicated concentrations of BHMC for 24 h. Cell lysates were probed with (**A**) β-PIX, (**B**) MMP-9, and (**C**) MT1-MMP and normalized to Glyceraldehyde-3-phosphate dehydrogenase (GAPDH) level. All data are the mean ± S.E.M of three independent experiments. * *p* < 0.05, ** *p* < 0.01 and *** *p* < 0.001, which is significantly different from the untreated group.

Previous studies have demonstrated that migration and invasion are key steps in cancer metastasis [33]. Matrix metalloproteinases (MMPs)-9 have been reported to also be linked with migration and invasion [33,34] and MT1-MMP was revealed to be associated with invasion specifically in the formation of invadopodia [35,36]. Similarly, β-PIX was found to be involved in migration and invasion [37] and in invadopodia formation of breast [38] and ovarian cancer cells [39]. BHMC treatment at a concentration of 12.5 µM significantly reduced the expression of MMP-9 (*p* < 0.01), MT1-MMP (*p* < 0.05), and β-PIX (*p* < 0.001) in MDA-MB-231 cells (Figure 4A–C), which suggests that this compound has the ability to prevent the migration and invasion of MDA-MB-231 especially in reducing the formation of invadopodia.

## 3. Discussion

Cancer metastasis is a selective event wherein the tumor cells only invade the surrounding that favors its growth once the cells detach from the primary tumor [40,41]. The steps involved in cancer metastasis include the detachment of the tumor cells from the primary tumor and invasion into surrounding tissue whereby the angiogenesis process also occurs simultaneously to supply nutrients and oxygen to the tumor cells [42]. Breast cancer is well-known for its ability to spread to other parts of the body [3]. In this study, we investigated the effects of BHMC on the proliferation, migration, and invasion of MDA-MB-231 breast cancer cells.

Cancer cells have undergone abnormal proliferation and dissociation from the tissue of origin to disseminate to other organs [43]. Proliferating cell nuclear antigen (PCNA) is a type of marker used to detect cell proliferation [44]. PCNA is involved in DNA replication and repair and cooperates as a cofactor to the DNA polymerase to prevent enzymes from separating from the DNA template by encircling the DNA [45]. Treatments of BHMC were chosen from 1.6 to 12.5 µM that showed cell viability of 80% and above. Thus, these concentrations were tested again by staining the PCNA antibody on MDA-MB-231 cells to confirm the effect of BHMC on the presence of PCNA in order to avoid interference resulting from the anti-proliferative effect of BHMC.

BHMC was observed to not affect the presence of proliferating cell nuclear antigen (PCNA) in MDA-MB-231 cells (Figure 1C). However, the presence of PCNA was observed in the cytoplasm of the cells (Figure 1B). PCNA is a well-known nuclear protein [44]. Its presence in the cytoplasm indicates that the protein was translocated from the nucleus to the cytoplasm [44]. It is suggested that the presence of PCNA in the cytoplasm may be involved in cytoskeleton regulation. One study demonstrated the binding of PCNA to cytoplasmic and membrane proteins, indicating that the interaction may be required in cancer migration [46]. Ultimately, more data needs to be collected to elucidate the function of cytoplasmic PCNA and cancer migration and invasion to provide new insights into the pathological improvement of cancer.

BHMC treatment was further studied to observe its effects on migration and invasion of MDA-MB-231 cells (Figure 2A–C). The ability of the tumor cells to migrate and invade into the surrounding tissue is the hallmark of tumor metastasis [47]. In the present study, treatment using BHMC has shown significant reduction in the percentage of migration and invasion of MDA-MB-231 cells (Figure 2A–C). Previous studies have reported curcumin to also prevent the invasion of MDA-MB-231 and MCF-7 breast cancer cells [25,48] and SCC-25 oral squamous cell carcinoma [24]. Besides that, a reduction in the number of cells forming invadopodia in the MDA-MB-231 cells upon all concentrations of BHMC treatment in this study in comparison to the untreated group was also observed (Figure 3). Invadopodia are thought to be formed and utilized by highly invasive cancer cells to degrade the matrix in the physiological environment to drive the invasion process [49]. A study conducted using safflower, a Chinese medicine, has also been performed to determine its effects on the formation of invadopodia in MDA-MB-231 cells [50]. This compound has also shown reduction in invadopodia formation. This is possibly due to the reorganization of the cytoskeleton and the reduction in the expression level of invadopodia-related proteins, which are p-Src and MMP-9, thus preventing the formation of invadopodia [50]. Therefore, it is plausible that one of the reasons for the reduction in the number of cells forming invadopodia is due to the interruption of cytoskeleton upon BHMC treatment. A compound called GM6001 was used to synchronize the formation of invadopodia [51]. As expected, there is absence of cells forming invadopodia when treated with GM6001, a broad range MMP inhibitor [51]. Invadopodia were not observed due to the inhibition of MMPs, but appeared again when the inhibitor was washed out [52].

In recent years, increasing evidence has shown that invadopodia recruit many signaling proteins and proteases, which either aim to promote actin assembly or to degrade the matrix [11]. In this study, for the first time we demonstrated that the treatment of BHMC successfully reduced the expression of β-PIX, MMP-9, and MT1-MMP in MDA-MB-231 cells (Figure 4A–C). It is therefore reasonable to speculate that the inhibition of invadopodia by BHMC may be partly due to the significant reduction in the expression levels of these invadopodia-related proteins. β-PIX is a guanine nucleotide exchange factor for Rac and Cdc42 and has been implicated in the regulation of cell adhesion and cell migration [12,53]. β-PIX has also been associated with invadopodia formation in breast cancer [38]. In a study conducted against a background of hypoxia, the β-PIX expression level was up-regulated upon expressing MDA-MB-231 cells with hypoxia and the number of cells with invadopodia decreased upon silencing of β-PIX [38]. Another study was also conducted to check whether or not the G protein is related to β-PIX and Src, a protein that is confined to invadopodia and shows the invasive migrating characteristics of tumor cells [54]. Src leads to the recruitment of β-PIX and activates the Rac protein in invadopodia [37]. It has been found that a G protein known as Gα_i2_ has been co-localized with Src and β-PIX in ovarian cancer cells, suggesting that β-PIX has prominently appeared in invadopodia in vigorously migrating cells [39]. Since BHMC reduced the expression of β-PIX at 12.5 µM, we postulate that the reduction in number of cells forming invadopodia is due to the treatment of BHMC that affects the β-PIX signaling pathway.

Other proteins investigated in this study were MMP-9 and MT1-MMP. MMP-9 has been thought to be related to cell migration and invasion by degrading the collagen and other matrix thus facilitating the metastasis of cancer cells [55,56,57]. A study demonstrated the role of cortactin in the production of invadopodia-associated MMPs: matrix metalloproteinase-2 (MMP-2), MMP-9, and MT1-MMP [36]. Surprisingly, the decrease in invadopodia actin puncta formation through the inhibition of MMPs is parallel to the silencing of cortactin, which implies that cortactin can stimulate the secretion of MMPs [36]. Hispolon, a Chinese traditional medicine, which is isolated from *Inonotus hispidus* and then identified in a number of traditional medicinal mushrooms, including *Phellinus linteus* and *Phellinus igniarius*, is being used to treat numerous types of cancers [58]. Hispolon hinders the invasion of MDA-MB-231 breast cancer cells by inhibiting the MMP-9 expression through the NF-κB pathway [58]. The findings indicate that the binding of MMP-9 is inhibited when NF-κB is inhibited. It is therefore postulated that NF-κB would be the upstream regulator of MMP-9 [58]. The treatment of BHMC had also reduced the expression level of MMP-9 in breast cancer cells in the present study. Therefore, it would be interesting to be able to investigate the MMP-9 association with this pathway downstream of BHMC treatment in the future.

On the other hand, curcumin has also been seen to reduce the expression of MMP-9 in astroglioma cells via inhibition of the PKC to MAPK pathway [59]. This is consistent with the results in the present study, whereby the treatment of BHMC reduced the expression level of MMP-9 at 12.5 µM (Figure 4B). Since BHMC is an analogue of curcumin, it may be plausible to speculate that the reduction in the migration and invasion may be, at least in part, working in the same pathway.

Membrane type-1 matrix metalloproteinase (MT1-MMP) has been associated with the degradation of the matrix in invadopodia formation [36]. The secretion of MT1-MMP has been directly correlated with another important protein in invadopodia formation, which is cortactin [60]. Cortactin is an invadopodia-related protein that recruits other additional proteins such as Arp 2/3, N-WASP, WASP-interacting protein (WIP), Extracellular signal-regulated (Erk) Kinase, and Myosin light-chain kinase (MLCK) to help in the actin assembly [61]. Cortactin has been shown to reduce the expression of matrix-degrading MMPs, which are MMP-2, MMP-9, and MT1-MMP suggesting that at the early stage of invadopodia formation, cortactin plays a pivotal role to deliver the MMPs before matrix degradation takes place [60]. Prior to these findings, the role of MT1-MMP on invadopodia formation were reported, whereby silencing MT1-MMP causes a significant decrease in invadopodia formation [36]. Recent studies have demonstrated the possible pathway that could regulate the appearance of MT1-MMP in invadopodia formation. The molecules involved in MT1-MMP trafficking and the contribution to breast cancer invasion is possibly due to the ARF6 and kinesin-1/KIF5B [13]. Therefore, the findings may shed new light into better understanding the cell type-specific marker of tumor invasiveness and to avoid side effects to non-cancerous cells [13]. In the present study, we observed that treatment of BHMC reduced the expression of MT1-MMP at 12.5 µM (Figure 4C). It has been reported that curcumin has also inhibited the invasion of the A549 lung cancer in MT1-MMP/MMP-2 pathway [62]. Moreover, the MT1-MMP expression level was also being suppressed upon curcumin treatment in prostate cancer cell lines [63]. Thus, we infer that the reduction in the invasiveness of breast cancer in the present study may be partly working in the same pathway, which is due to the reduction of the MT1-MMP expression.

It can also be observed that the BHMC treatment was able to inhibit the formation of invadopodia starting from 1.6 µM. However, BHMC is only able to inhibit the other parameters, especially the protein expression of β-PIX, MMP-9, and MT1-MMP, at 12.5 µM. We speculate that only low concentrations of BHMC are required to impede the protrusions but a higher concentration of BHMC is needed to inhibit the protein expression and the invasion of whole cells. Perhaps, treatment of BHMC at low concentration interrupts the structural or functional components that contribute to the formation of invadopodia on a single cell in the 2D gelatin degradation assay [50].

This study demonstrates an important link between BHMC and the invasiveness of MDA-MB-231 breast carcinoma cells by significantly inhibiting invadopodia formation and reducing invasion/invadopodia-related protein expressions such as β-PIX, MMP-9, and MT1-MMP. Treatment of BHMC may also be connected with Cdc42 and Rac1, proteins that are involved mainly in migration and invasion; thus, aiding in inhibiting the migration and invasion in MDA-MB-231 cells. Previous studies have demonstrated that curcumin has also inhibited Cdc42 and Rac1 and prevented the migration and invasion of lung cancer [64,65]. Thus, with more validation work on BHMC, the compound can potentially be used as an anti-metastatic therapeutic agent in breast cancer treatment in the future.

## 4. Materials and Methods

### 4.1. Reagents

Antibiotics (5000 U/mL penicillin and 5000 μg/mL streptomycin), fetal bovine serum (FBS), and Dulbecco’s Modified Eagle Medium (DMEM) were purchased from Gibco (Gaithersburg, MD, USA). Anti-Cool/ β-PIX, anti-MMP-9, and anti-MT1-MMP were purchased from Cell Signaling Technology (Danvers, MA, USA). Alexa Fluor 488 Phalloidin, Alexa Fluor 568 goat anti-mouse, Rhodamine phalloidin Oregon green 488 gelatin, Prolong Gold anti-fade mounting reagent, and Hoechst 33342 were obtained from Invitrogen (Carlsbad, CA, USA). Anti-Proliferating cell nuclear antigen (PCNA) was obtained from Thermo Fisher Scientific (Waltham, MA, USA). Paraformaldehyde, 25% glutaraldehyde and sodium borohydride were obtained from Sigma Aldrich (St. Louis, Missouri, USA). HRP-conjugated goat anti-rabbit Ig-G and anti- Glyceraldehyde-3-phosphate dehydrogenase (GAPDH) were purchased from Santa Cruz Biotechnology (Santa Cruz, CA, USA). All the other reagents for the western blot analysis were from Nacalai Tesque (Kyoto, Japan) and Amresco (Solon, OH, USA). 3-(4,5-Dimethylthiazol-2-yl)- 2,5-diphenyltetrazolium bromide (MTT), bovine serum albumin (BSA) and dimethyl sulfoxide (DMSO) were also purchased from Amresco. The polyvinylidene fluoride (PVDF) membrane and bicinchoninic acid (BCA) protein determination kit were purchased from Merck Milipore (Burlington, MA, USA). Enhanced chemiluminescene reagent was purchased from Advansta (Menlo Park, CA, USA). Rat-tail collagen type I was purchased from BD Corning (Corning, NY, USA). Protein ladders were purchased from GeneDirex (Taoyuan, Taiwan). 

### 4.2. Cell Culture

MDA-MB-231 cells were obtained from the American Type Culture Collection (ATCC, Manassas, VA, USA). Cells were cultured in DMEM with 10% fetal bovine serum (FBS), 100 U/mL penicillin, and 100 μg/mL streptomycin in a 5% CO_2_ incubator at 37 °C. These adherent cells were grown until 80–90% confluent. For corresponding experimental treatments, the complete growth media was removed and the cells were washed twice with 5 mL phosphate buffer saline (PBS) followed by the addition of 0.25% Trypsin/EDTA to remove them from the plate. The changes in cell morphology were observed using an inverted phase contrast microscope (Leica Microsystems, Wetzlar, Germany).

### 4.3. MTT Assay

The cells were seeded in triplicate in a 96-well plate at a seeding density of 1 × 10^5^ cells/mL in 100 µL 10% DMEM followed by 24 h of incubation. The cells were treated with various concentrations of BHMC, which ranged from 0, 1.6, 3.1, 6.3, 12.5, 25, 50, and 100 µM and prepared in 10% DMEM containing 0.1% DMSO. The cells for normal control (untreated, UT) and vehicle control (considered as 0 concentration) were added with 10% DMEM and 0.1% DMSO, respectively. The concentration of DMSO was maintained at 0.1% throughout all the experiments. Following treatments, the cells were further incubated for 24 h. Ten microliters (5 mg/mL) of MTT were added and the plate was incubated for another 3 h prior to the addition of 100 uL/well of DMSO to dissolve the blue formazan crystals formed. The absorbance was measured at a wavelength of 570 nm with a reference length of 620 nm using a microtitre reading plate machine (Versamax, San Jose, CA, USA). The experiment was repeated at least for three independent experiments. Fifty percent DMSO was used as positive control. The method was adapted and modified from [27].

### 4.4. Proliferation Assay

The MDA-MB-231 cells were seeded on the coverslips in a 24-well plate at 2 × 10^4^ cells/mL and incubated overnight. Treatment with BHMC was prepared in 10% DMEM and incubated for 24 h. The cells were fixed with 4% paraformaldehyde for 20 min and washed three times with PBS. Then, the cells were permeabilized with 0.2% Triton X-100 for 5 min followed by three times washing with PBS and blocking with 3% BSA for 30 min. The cells were washed three times with PBS 5 min each and stained with anti-PCNA monoclonal antibody (Invitrogen, Cat No.: MA5-11358) at a dilution of 1:100 in 3% BSA for 2 h at room temperature in dark humidified condition. The coverslips were washed three times with PBS 5 min each and proceeded with staining of Alexa Fluor 568 goat anti-mouse secondary antibody diluted in 3% BSA at a dilution of 1:200 for 1 h together with 488 phalloidin. The coverslips were washed three times with PBS for 5 min each and then mounted onto the slide with Prolong Gold anti-fade mounting reagent (Invitrogen). The images were captured to compare between the treated and untreated cells to observe the difference in the nucleus appearance that represented the PCNA staining. The experiment was repeated at least for three independent experiments. The method was adapted and modified from [48].

### 4.5. Scratch Migration Assay

An in vitro scratch migration was conducted to assess cell motility. The method was adapted and modified from [66]. Briefly, MDA-MB-231 cells were grown in a 6-well plate (2 × 10^5^ cells/well). Upon cell confluence, a scratch was made using a yellow tip with approximately 1 mm diameter followed by washing the cells twice with PBS. Treatments with non-cytotoxic concentrations (0, 1.6, 3.1, 6.3, and 12.5 µM) of BHMC in DMEM were added accordingly to the labelled well and were further incubated for 24 h. A line was marked on the lid of the well to indicate the position of the images captured. The images were captured at 0 h (right after the addition of the treatment) and 24 h using a Dino Eye application attached to the inverted microscope (Leica Microsystems) at a magnification of 40×. At least for three independent experiments were performed. The percentage of migration of MDA-MB-231 cells was calculated by = [((distance of the scratch at 0 h − distance of scratch at 24 h)/ distance of the scratch at 0 h) × 100].

### 4.6. Transwell Migration and Invasion Assays

The migration and invasion capability of MDA-MB-231 cells was determined using 8 µm transwell inserts from BD Corning Inc. The method was adapted and modified from [67]. In brief, the cells were seeded at 1 × 10^5^ cells/well in the upper part of the insert filled with 200 µL of BHMC containing 0.5% FBS. For the lower part, the chamber was filled with 600 µL DMEM containing 10% FBS to act as chemoattractant. For the transwell invasion assay, the inserts were coated with 50 µg/mL rat-tail type I collagen (BD Corning). The cells were incubated at 37 °C for 24 h for both migration and invasion assay. Non-migrating and non-invading cells were removed from the upper chamber. Migrating and invading cells were stained with 0.2% crystal violet in 20% methanol. The quantification of the cells migrated and invaded was done by dissolving the dye with acetic acid solution, and the absorbance was read at 570 nm using a microtitre reading plate machine (Versamax). At least three independent experiments were conducted.

### 4.7. Gelatin Degradation Assay

The procedure was adapted from [38,68]. Ethanol-washed 12 mm round coverslips were coated with Oregon Green 488 gelatin (Invitrogen) at 0.2 mg/mL with PBS containing 2% sucrose for 10 min in the dark. After that, 100 µL of 0.5% pre-chilled glutaraldehyde in PBS was used to crosslink the gelatin for 15 min. The coverslips were transferred into a 24-well plate with the coated surface up and washed three times with PBS. One milliliter of 5 mg/mL of sodium borohydride was added into the 24-well plate and incubated for 3 min. The coverslips were once again washed with PBS three times followed by sterilization with 70% ethanol. MDA-MB-231 cells seeded in a 6-well plate at 5 × 10^4^ cells/mL were treated with various concentrations of BHMC for 24 h. Following treatment, the cells were trypsinized and re-seeded (2 × 10^4^ cells/mL) onto gelatin-coated coverslips and incubated for 3 h. The cells were fixed with 4% paraformaldehyde for 20 min followed by three times washing with PBS. The cells were permeabilized with 0.2% Triton X-100 in PBS for 5 min and stained with Rhodamine phalloidin (Invitrogen) diluted in 3% BSA in PBS and incubated in a dark humidified environment for 1 h. The cells were counter-stained with Hoechst 33342 (Invitrogen) to stain the nucleus. The coverslips were then mounted with ProLong Gold anti fade (Invitrogen). The slides were observed for the formation of invadopodia. The number of cells forming invadopodia were counted for 100 cells and converted into a percentage. The experiment was repeated at least for three independent experiments. The comparison between the treated and untreated cells was done statistically.

### 4.8. Immunoblotting

MDA-MB-231 cells were treated with various concentrations of BHMC for 24 h. Whole cell lysate was used to stain for anti-Cool/β-PIX, anti-MMP-9, and anti MT1-MMP. The protein quantification was done using a BCA assay kit (Merck). Up to 20 µg protein of each treatment was loaded into 10% SDS-polyacrylamide gel and blotted on a 0.45 µm polyvinylidene difluoride (PVDF) membrane (Merck). The membranes were then blocked in 5% BSA in Tris buffered saline (TBS)-Tween 20 for 1 h and incubated overnight with goat anti-rabbit polyclonal Cool/β-PIX and MMP-9 (1:1000) and goat anti-rabbit monoclonal MT1-MMP antibody (1:1000) in TBS-Tween containing 5% BSA. For GAPDH blotting, the membranes were blotted with anti-rabbit GAPDH (1:2000) for 1 h. After washing three times with TBS-Tween, the membrane was hybridized with HRP-conjugated goat anti-rabbit secondary antibody (1:5000) in TBS-Tween containing 5% BSA for 1.5 h and washed three times with TBS-Tween. The membrane was incubated with ECL reagent and viewed under chemiluminescence using a gel documentation system (Vilber Lourmat, Vilber, Marne-la-Vallée, France). Band intensities were quantified with an Image J Java-based image processing program and normalized by comparison to GAPDH. The experiment was repeated at least for three independent experiments.

### 4.9. Statistical Analysis

All data was presented as means ± S.E.M of three separate experiments performed in triplicate. GrapPad Prism software version 5.0 (GraphPad Software, La Jolla, CA, USA) was used to determine the differences between groups by using One-Way Analysis Of Variance (ANOVA) followed by post-hoc Dunnett’s test. A *p*-value of less than 0.05 was considered to be significant.

## 5. Conclusions

This study has demonstrated that BHMC at a non-cytotoxic concentration of 12.5 µM affects the invasiveness of MDA-MB-231 human breast cancer cells. The migration and invasion of MDA-MB-231 human breast cancer cells has been hindered upon treatment with BHMC at 12.5 µM. More importantly, treatment of BHMC has significantly reduced the number of MDA-MB-231 cells forming invadopodia. Furthermore, this study is the first report to suggest the molecular targets involved in the BHMC mechanism for controlling the invasiveness of MDA-MB-231 human breast cancer cells, whereby treatment of BHMC at 12.5 µM reduced the expression level of β-PIX, MMP-9, and MT1-MMP. These molecular targets are implicated in the invasion of breast cancer, especially in the formation of invadopodia.
